# Supplementary material for: Substrate Recognition and Autoinhibition in the Central Ribonuclease RNase E
Source: Mol Cell. 2018 Oct 18;72(2):275–285.e4. doi: 10.1016/j.molcel.2018.08.039 (PMC6202311; doi:10.1016/j.molcel.2018.08.039)
Supplement: Document S1. Figures S1–S3 [file mmc1.pdf]

Molecular Cell, Volume *72*

Supplemental Information

Substrate Recognition and Autoinhibition in the Central Ribonuclease RNase E

Katarzyna J. Bandyra, Joanna M. Wandzik, and Ben F. Luisi

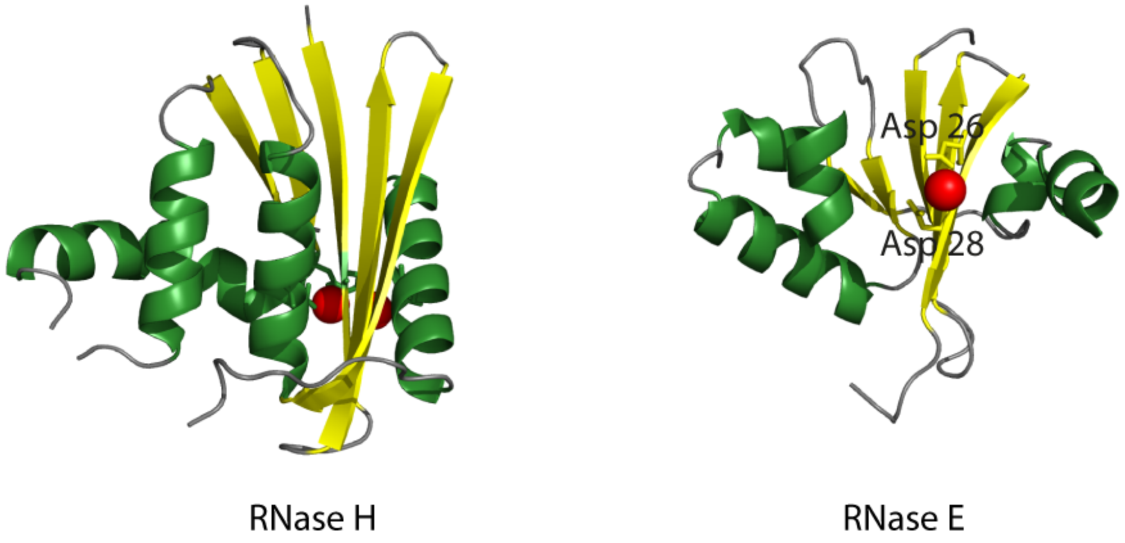

Figure S1. Related to Figure 2. Comparison of E. coli RNase H (1G15) and RNase H domain from RNase E-MicC structure. Bound metal ions (two manganese ions for RNase H and one magnesium ion for RNase E) are shown as red spheres. The acidic residues coordinating metal ions are shown as sticks.

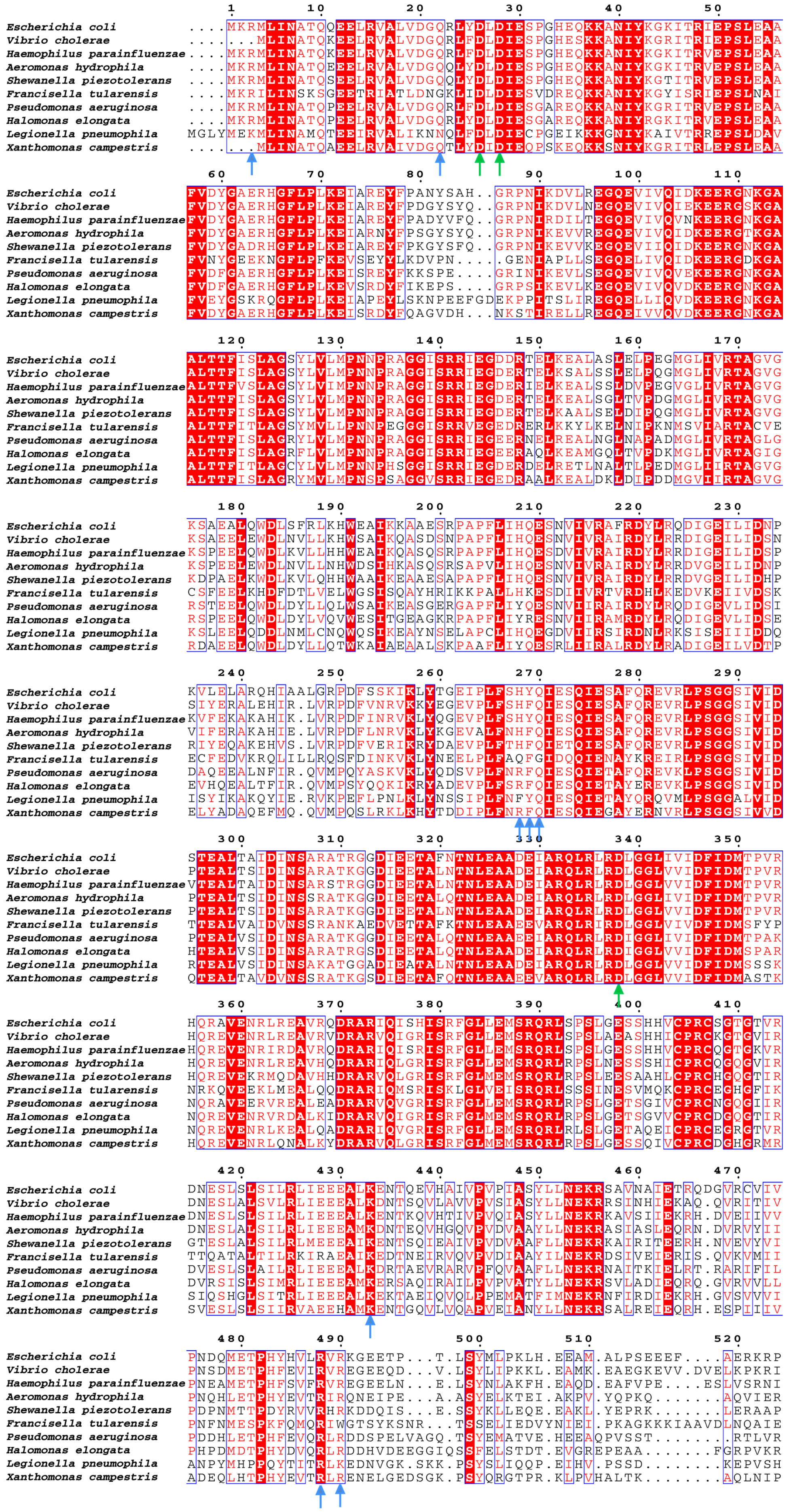

Figure S2. Related to Figure 2. Alignment of RNase E catalytic domain from representative species of γ-Proteobacteria. Green arrows mark residues coordinating magnesium ion in the newly identified magnesium binding site; blue arrows mark residues implicated in interaction with RNA structural elements based on the crystal structure of the RNase E NTD/RprA complex.

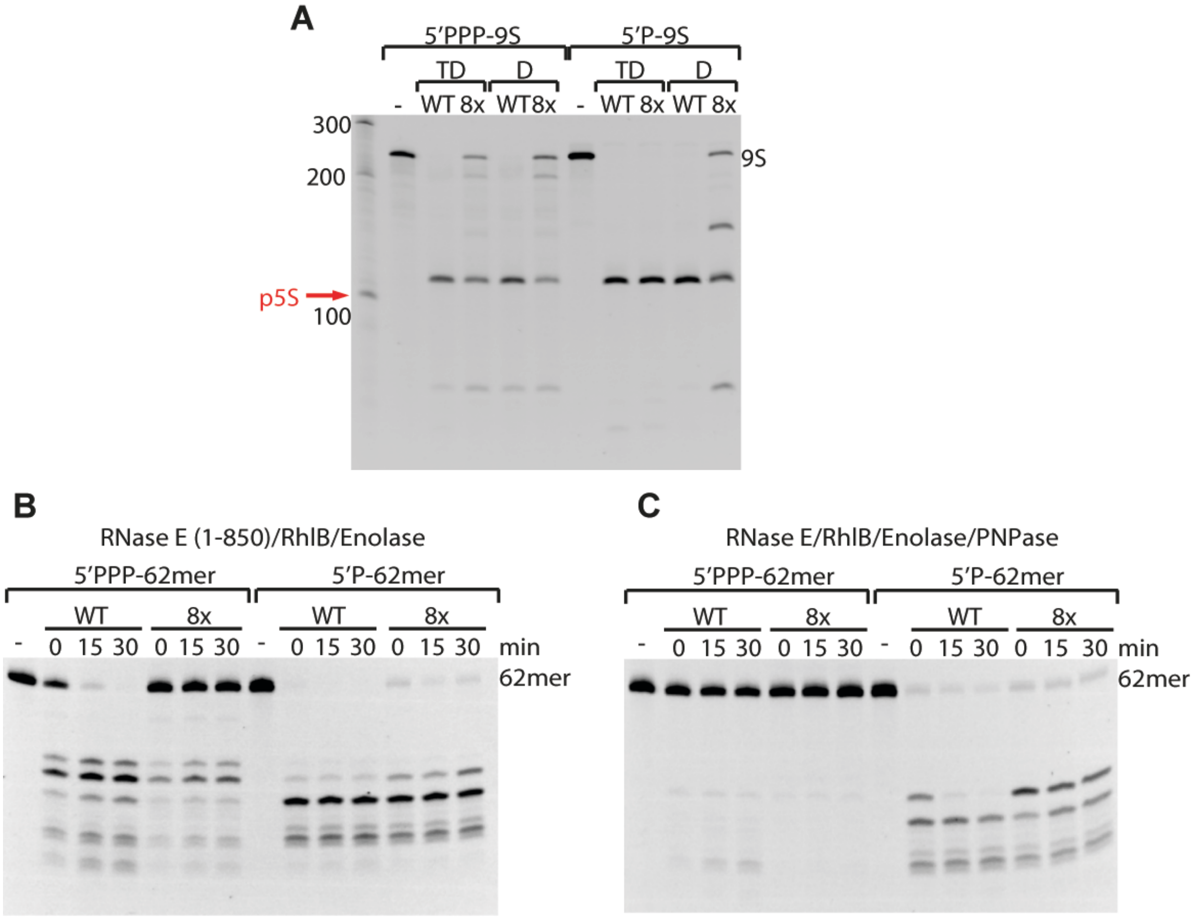

Figure S3. Related to Figure 5. The duplex interaction surface contributes to RNA binding and cleavage. A) 9S rRNA (200 nM) with 5' tri- or monophosphate processing by 100 nM RNase E (1-850)/RhlB/Enolase (TD, truncated degradosome) and 15 nM full length RNase E/RhlB/Enolase/PNPase (D, degradosome), wild type and 8x mutants (R3H, Q22D, H268S, Y269F, Q270D, K433N, R488Q, R490Q). C) 62-mer RNA (5µM) with 5' tri- or monophosphate processing by 200 nM RNase E (1-850)/RhlB/Enolase and 32 nM full length RNase E/RhlB/Enolase/PNPase, wild type and 8x mutants.
