# Supplementary material for: Substrate Recognition and Autoinhibition in the Central Ribonuclease RNase E
Source: Mol Cell. 2018 Oct 18;72(2):275–285.e4. doi: 10.1016/j.molcel.2018.08.039 (PMC6202311; doi:10.1016/j.molcel.2018.08.039)
Supplement: Document S2. Article plus Supplemental Information [file mmc2.pdf]

Article

Molecular Cell

Substrate Recognition and Autoinhibition in the Central Ribonuclease RNase E

Graphical Abstract

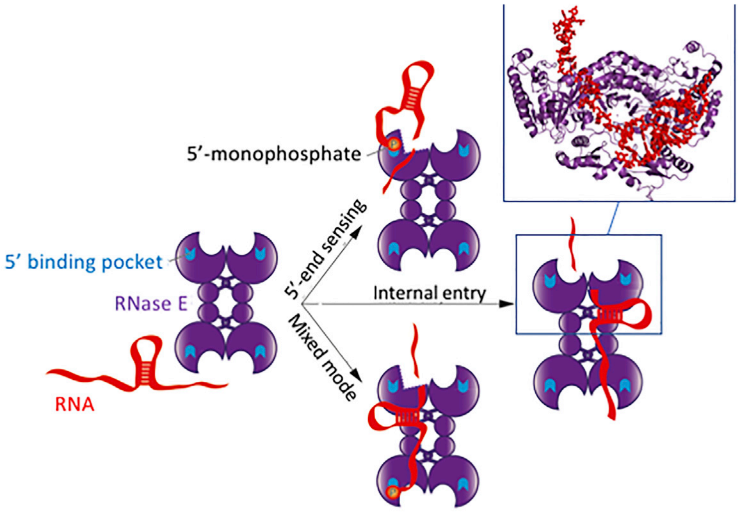

RNase E modes of substrate recognition

Highlights

- RNase E recognizes RNA secondary structure
- Signature on the substrate 5′ end recognizes and activates RNase E
- RNase E intrinsic activity is repressed by a conserved autoinhibition motif

Authors

Katarzyna J. Bandyra, Joanna M. Wandzik, Ben F. Luisi

Correspondence

kjb69@cam.ac.uk (K.J.B.), bfl20@cam.ac.uk (B.F.L.)

In Brief

RNase E is a central ribonuclease of RNA metabolism and post-transcriptional control of gene expression. Through structural and functional analyses, Bandyra et al. reveal how the enzyme activity can be favored by recognition of the 5′ end and RNA secondary structure. The data also reveal a conserved autoinhibitory motif that suppresses RNase E intrinsic activity.

Bandyra et al., 2018, Molecular Cell *72*, 275–285
October 18, 2018 © 2018 The Author(s). Published by Elsevier Inc.
https://doi.org/10.1016/j.molcel.2018.08.039

CellPress

Substrate Recognition and Autoinhibition in the Central Ribonuclease RNase E

Katarzyna J. Bandyra,[1,*] Joanna M. Wandzik,[1,2] and Ben F. Luisi[1,3,*]
[1]Department of Biochemistry, University of Cambridge, Tennis Court Road, Cambridge CB2 1GA, UK
[2]Present address: European Molecular Biology Laboratory, Grenoble Outstation, 71 Avenue des Martyrs, CS 90181, 38042 Grenoble Cedex 9, France
[3]Lead Contact
*Correspondence: kjb69@cam.ac.uk (K.J.B.), bfl20@cam.ac.uk (B.F.L.)
https://doi.org/10.1016/j.molcel.2018.08.039

SUMMARY

The endoribonuclease RNase E is a principal factor in RNA turnover and processing that helps to exercise fine control of gene expression in bacteria. While its catalytic activity can be strongly influenced by the chemical identity of the 5′ end of RNA substrates, the enzyme can also cleave numerous substrates irrespective of the chemistry of their 5′ ends through a mechanism that has remained largely unexplained. We report structural and functional data illuminating details of both operational modes. Our crystal structure of RNase E in complex with the sRNA RprA reveals a duplex recognition site that saddles an inter-protomer surface to help present substrates for cleavage. Our data also reveal an autoinhibitory pocket that modulates the overall activity of the ribonuclease. Taking these findings together, we propose how RNase E uses versatile modes of RNA recognition to achieve optimal activity and specificity.

INTRODUCTION

Ribonuclease E (RNase E) is a metal-dependent hydrolytic enzyme that cleaves polymeric ribonucleic acid internally and serves as a central element in RNA metabolism of diverse bacteria (Mackie, 2013). The enzyme plays a role in regulation of gene expression both directly through transcript degradation and indirectly by processing of regulatory RNAs (Chao et al., 2017). RNase E cleavage is observed in processes such as messenger RNA degradation, transfer RNA and ribosomal RNA maturation, generation of small regulatory RNA (sRNA), and sRNA-mediated gene silencing (Mackie, 2013; Dar and Sorek, 2018).

RNase E forms a multi-enzyme complex called the RNA degradosome, together with the DEAD-box RNA helicase B (RhlB), the glycolytic enzyme enolase, and the phosphorolytic exonuclease polynucleotide phosphorylase (PNPase) (Carpousis, 2007). RNase E can be considered as a core component of the degradosome since it contributes both functional and structural roles in this assembly. Composed of 1,061 amino acids, the enzyme can be divided into two parts of almost equal mass: the N-terminal and C-terminal domains (NTD and CTD, respectively). The RNase E CTD, which is mostly unstructured and has varied rapidly in evolution (Aït-Bara et al., 2015; Callaghan et al., 2004; Marcaida et al., 2006), forms a scaffold for degradosome assembly as it harbors binding sites for protein partners, as well as microdomains responsible for RNA binding and membrane association (Aït-Bara et al., 2015; Bandyra et al., 2013; Khemici et al., 2008). The NTD of RNase E (1–529) is highly conserved and harbors the catalytic activity of the enzyme. Crystal structures of the NTD have revealed its domain organization and provided insight into the catalytic mechanism (Callaghan et al., 2005; Koslover et al., 2008). RNase E forms a tetramer in which the protomers are arranged as a dimer of dimers and accommodate an RNA substrate at the active site formed by the interface of two subunits of the principal dimer unit (Figure 1A). The catalytic site is located in a DNase I-like domain, where conserved acidic groups D303 and D346 coordinate the magnesium ions that assist in the hydrolytic attack of single-stranded RNA. One of the domains in the NTD has an RNase H fold, although this domain has not retained the key catalytic residues of the conventional active site found in the RNase H family.

Given the pivotal role of RNase E in RNA processing, turnover, and RNA-mediated regulation, many of the enzyme cleavage preferences are expected to be reflected in the characteristics of its substrates. One salient signature of RNase E cleavage sites is that they are AU-rich and single-stranded (Clarke et al., 2014; Huang et al., 1998; McDowall et al., 1994, 1995), with strong preference for U at position +2 with respect to the scissile phosphate (Chao et al., 2017). This signature is in accord with the crystallographic data, as the catalytic site cannot fit double-stranded substrates, and U+2 is predicted to make favorable and conserved interactions with the NTD that help to orientate the scissile phosphate for hydrolytic attack (Chao et al., 2017).

A striking feature of RNase E is that its activity on certain substrates can be increased if the 5′ end harbors a monophosphate group (Mackie, 1998). Crystallographic studies show that the terminal phosphate is recognized by a hydrogen bonding network involving R169 and T170 within the 5′ sensor domain, and this interaction is proposed to favor a closed conformational state that boosts the enzyme activity (Callaghan et al., 2005). Comparison of crystal structures of the apoenzyme and holoenzyme, in which an RNA substrate analog is bound, shows that RNase E can adopt an open form in the apo state that closes upon

© 2018 The Author(s). Published by Elsevier Inc.
This is an open access article under the CC BY license (http://creativecommons.org/licenses/by/4.0/).

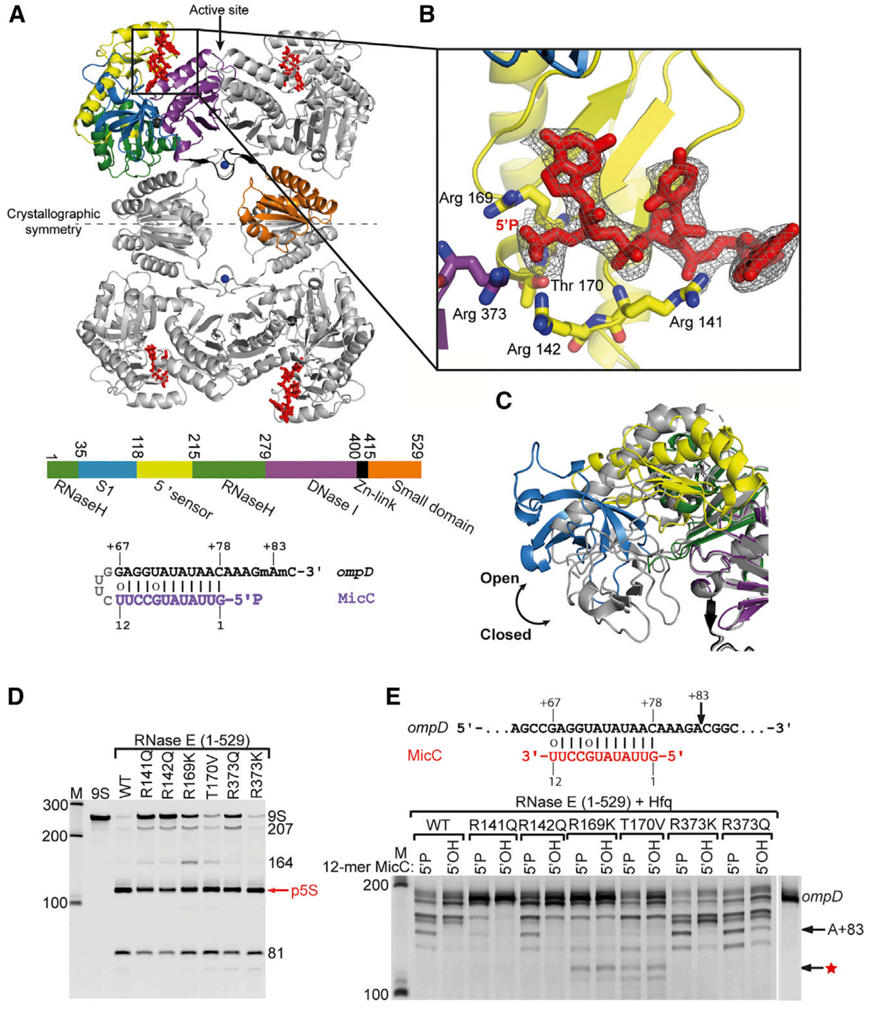

Figure 1. The Structure of RNase E with a Fragment of MicC sRNA Identifies Residues Involved in 5′ End Recognition

(A) RNase E (1–529)-MicC quaternary structure. The individual subdomains of one protomer are color coded as indicated in the coding bar. Bound RNA fragment (red), interacting with 5′ sensor and S1 domains, is colored on each subunit. The schematic of RNA substrate used for crystallization is shown in the bottom of the panel. The lines indicate predicted pairings and the circles wobble-pairs.

(B) Contacts to the 5′ end of the RNA. The ternary and quaternary structural changes as well as the 5′ end sensor domain contacts to the RNA are corroborated by another structure of RNase E at 3.5–3.7 Å resolution crystallized in a different space group with 1.5 tetramers in the asymmetric unit (data not shown). The anneal-omit map (Fo-Fc coefficients) was calculated using the final coordinates and contoured at 2.3 sigma.

(C) Tertiary structural changes associated with closed (2c4R, gray) and open (color coded as in A) states for a protomer.

(D) The role of the amino acids in- and outside of the 5′ binding pocket in processing 9S rRNA. The red arrow indicates 5S precursor (p5S), the final product of 9S processing.

(E) The role of the amino acids in- and outside of the 5′ binding pocket in MicC-mediated *ompD* degradation. RNase E (1–529) wild-type (WT) and mutants (R141Q, R142Q, R169K, T170V, R373Q, and R373K) were evaluated with 200 nM 9S or 200 nM *ompD* in the presence of 300 nM 12-mer MicC. For each enzyme, the concentration used was 200 nM for 9S assays and 150 nM for MicC/*ompD* assays. The size markers are in the left lanes (M). The black arrow indicates the 153 nt long product of the +83 cleavage of *ompD*, which is the *in vivo* observed MicC-guided cleavage. The star indicates a new cleavage site observed for RNase E (1–529) R169K and T170V.

substrate binding (Callaghan et al., 2005; Koslover et al., 2008). This movement enables proper orientation of substrate for catalysis and is favored by the interaction of the 5′ end with the sensing pocket. While the 5′ sensing pocket can accommodate 5′ triphosphate, the enzyme cannot close upon the substrate due to steric hindrance, and in consequence the catalytic activity is impeded (Callaghan et al., 2005). Studies with short oligonucleotides indicate that the apparent boost in catalytic efficiency for substrates with 5′ monophosphate compared to those with 5′ hydroxyl group arises principally from the reduced Michaelis-Menten parameter, K_m (Kime et al., 2010), suggesting that the effect of 5′ end sensing is mostly to contribute to substrate binding.

A second, potentially large class of RNase E substrates exists for which the enzyme activity is not strongly affected by the chemical status of the 5′ end (Clarke et al., 2014; Kime et al., 2010, 2014). The existence of this class has led to the hypothesis of two potential pathways for substrate recognition by RNase E: a 5′ end-dependent mode that relies on 5′ monophosphate recognition described above and an internal entry mode (Bouvier and Carpousis, 2011). The internal entry pathway (also known as 5’ bypass) could rely in part on interactions of RNA substrates with the NTD and arginine-rich segments present in the CTD (Callaghan et al., 2004; Kaberdin et al., 2000). As RNase E is an essential enzyme in *E. coli*, the importance of those two pathways was tested *in vivo* by deactivating them individually and in combination. Strains harboring either a crucial point mutation in the phosphate binding pocket (R169Q) or with deleted CTD were viable (Garrey et al., 2009), suggesting that RNase E is still active when the terminal phosphate recognition is impeded or the C-terminal RNA-binding microdomains are removed. On the other hand, combining the mutations appeared to be lethal for *E. coli* strains (Garrey and Mackie, 2011). This suggests that two different ways of recognizing RNA substrates exist and can compensate for each other. On the other hand, these pathways are not necessarily mutually exclusive and may work in a cooperative manner.

Early studies of processing of complex substrates by RNase E indicate a role for secondary structure recognition by the enzyme. For example, the 9S gene product is cleaved by RNase E twice to yield the 5S precursor, which is further trimmed

by nucleases to give the mature 5S ribosomal RNA (Christiansen, 1988; Cormack and Mackie, 1992; Ghora and Apirion, 1978). Evidence indicates that a secondary structure in the 5′ domain of 9S is essential for its recognition by RNase E (Cormack and Mackie, 1992). More recently, high-throughput sequencing analysis on a transcriptome-wide scale in *E. coli* revealed that, in many mRNAs that are RNase E substrates, a stem-loop is present upstream of the cleavage site (Del Campo et al., 2015). These findings indicate that structural motifs in RNA substrates might be crucial for recognition by RNase E to help align single-stranded regions for cleavage.

Here we present crystal structures of the catalytic domain of RNase E in complex with RNA that illuminate details of both 5′ end sensing and internal processing modes of the enzyme. NTD co-crystallized with a fragment of the regulatory RNA MicC reveals interactions consolidating 5′ end recognition, and we demonstrate the importance of these contacts *in vitro*. The structure also reveals an electronegative cluster that, when mutated, tremendously boosts enzyme activity. We also present the crystal structure of the complex of RNase E with full-length RprA, an sRNA (106 nt in *Salmonella*) (Chao et al., 2017; Majdalani et al., 2002). RprA has biological roles in activation of gene expression of *rpoS* and *ricI* by disrupting self-inhibitory motifs in their transcripts (Majdalani et al., 2002; Papenfort et al., 2015; Soper et al., 2010). RprA contains three stem-loop motifs with an RNase E cleavage site within one of the loop regions. Cleavage at that site releases a shorter form of the sRNA. The crystal structure of the RprA/RNase E complex reveals the interaction site for a stem-loop structure, and mutagenesis analysis illustrates its role in the internal entry mode of the enzyme. Taken together, our structural and functional data present a comprehensive model for different modes of substrate recognition by RNase E.

RESULTS

Co-crystal Structure of RNase E (1–529) with RNA Substrate Identifies Residues Contributing to 5′ Sensing

In an effort to structurally characterize the mechanism of 5′ end sensing by RNase E and its interaction with sRNA, the catalytically inactive RNase E (1–529) D303R, D346R was purified and used in co-crystallization trials with sRNA bearing a 5′ monophosphate. The oligoribonucleotide comprises the MicC seed region connected through a tetranucleotide loop with a fragment of the *ompD* target encompassing the seed complementary sequence and the RNase E cleavage site (Figure 1A, bottom panel; Pfeiffer et al., 2009). Although catalytically inactive RNase E was used, to further ensure lack of residual RNA degradation, the nucleotides at the +82 and +83 positions with respect to the translation start site were modified with O2′-methyl groups to prevent cleavage (Figure 1A, bottom panel, annotated as m). Crystals of the protein:RNA complex were obtained, and an X-ray diffraction dataset was collected to 3.0 Å resolution. The structure was solved by molecular replacement (using PDB: 2BX2 as the reference model), and the solution provided clear electron density for all protein domains (Table 1). An RNA fragment of 2 to 3 bases could be modeled with a good fit to the density at each of the two independent 5′ sensor domains in the crystallographic asymmetric unit (Figure 1A). Given that the 5′ sensor domain is known to bind the 5′ end of RNA substrates, the RNA density was modeled as the 5′ end of MicC (Figure 1B). The ribonuclease assumes an "open" state quaternary structure, in which the S1/5′ sensor domains are rotated away from the active sites, where no RNA is bound (Figure 1C); however, the conformation is comparatively more closed than the apo state.

The 5′ monophosphate is bound in the 5′ sensing pocket, and the RNA forms additional contacts with RNase E (Figure 1B) that were not seen in the previous structure of the "closed" state, where the substrate is bound at the active site. For example, the phosphate backbone is engaged in hydrogen bonding interactions with R373 and R142 in addition to R169 and T170 identified earlier as the core of the 5′ sensing pocket (Callaghan et al., 2005). R141 also contacts the RNA backbone and supports the second base from the 5′ end (Figure 1B).

The roles of these newly identified contacts in sRNA-mediated target recognition were tested by mutagenesis. Conservative substitutions were introduced for the chosen residues: R141Q, R142Q, R373K, and R373Q; additionally, we also mutated residues that were earlier found to impact 5′ end sensing: R169K and T170V. We first tested the impact of the mutants on processing of 9S RNA, which earlier studies have shown is cleaved twice by RNase E to yield the precursor for ribosomal 5S RNA (Christiansen, 1988; Cormack and Mackie, 1992; Ghora and Apirion, 1978). All of the RNase E variants were capable of correctly processing 9S RNA into the 5S rRNA precursor, indicating that the mutations do not abrogate the overall enzyme specificity (Figure 1D), regardless of any influence they exercise on overall enzyme activity.

The same set of mutants was next tested for activity in target cleavage guided by sRNA. For the cleavage assay, the isolated seed region of MicC (12-mer MicC, 300 nM) and its target mRNA *ompD* (200 nM) were used. MicC was shown to guide RNase E cleavage of *ompD* at position +83 of the coding region of mRNA (in relation to the translation initiation; Bandyra et al., 2012; Pfeiffer et al., 2009), which results in the accumulation of an RNA fragment of 153 nt with the RNA substrate used in this study. Although all the mutants were active against the sRNA-mRNA pair, they performed differently (Figure 1E). R373 mutants seem to be slightly more active than wild-type (WT), especially toward the mRNA fragment in the presence of the 5′ OH sRNA: R373K cleaved about 93% and 88% of *ompD*, R373Q 89% and 83%, and WT 85% and 74%, in the presence of 5′P and 5′OH MicC, respectively. Interestingly, the R141Q mutant recognizes the substrate in the presence of 5′ monophosphorylated MicC, but its activity is very low, and it cleaved roughly 2% of the total substrate in the presence of 5′P MicC 12-mer compared to WT enzyme during the same time interval. The R141 side chain interacts with the RNA backbone at the entry to the 5′ binding pocket and therefore likely helps to orient the sRNA-mRNA duplex in a way that favors cleavage or aids unwinding the sRNA-mRNA duplex to align both molecules optimally on the enzyme. RNase E R142Q was less active, but still capable of 5′ monophosphate activation, cleaving about 68% and 42% of *ompD* in the presence of 5′P and 5′OH MicC 12-mer, respectively.

None of the four mutants, R141Q, R142Q, R373Q, or R373K, seem to affect the recognition of the 5′ end of the RNA, and

Table 1. Crystallographic Data and Refinement Parameters

Components	RNase E (1–529) D303R, D346R + MicC	RNase E (1–510) D303R, D346R + RprA
Resolution range (Å)	120.4–3.0 (3.11–3.00)	95.8–3.95 (4.19–3.95)
Space group	C2	$P6_1$
Unit cell (Å)	91.619 122.562 122.195; $\beta = 99.77^\circ$	110.630 110.630 466.02
Total reflections	50,945 (4,562)	270,611
Unique reflections	26,464 (2,488)	28,217 (4,519)
Multiplicity	1.9 (1.8)	9.6 (9.9)
Completeness (%)	99.20 (93.53)	100.0 (100.0)
Mean I/sigma(I)	14.04 (2.04)	8.3 (2.3)
Wilson B-factor	78.8	145.1
R-merge	0.051 (0.513)	0.150 (1.043)
R-measure	0.072	0.169 (1.111)
CC1/2	0.997 (0.587)	0.975 (0.501)
R-work	0.1937 (0.3516)	0.2747 (0.3655)
R-free	0.2445 (0.3810)	0.2962 (0.3666)
Number of non-hydrogen atoms	8,213	16,135
Atoms in macromolecules -protein/RNA	8,102/109	15,629/504
ligands (ions)	1 Zn^{++}, 1 Mg^{2+}	2 Zn^{2+}
water	0	0
RMS(bonds)	0.014	0.005
RMS(angles)	1.86	1.01
Ramachandran favored (%)	97.25	96.14
Ramachandran outliers (%)	0.0	0.21
Clashscore (MOLPROBITY)	2.38	1.71
Average B-factor ($Å^2$)	89.8	241.9
macromolecules ($Å^2$); protein/RNA	89.6/101.6	241/254
ligands ($Å^2$)	109.7	116.5
Estimated coordinate error (Å); maximum-likelihood based/Luzzati plot	0.60/0.60	0.66/1.34
PDB code	5F6C	6G63

even those with very low overall activity are still stimulated by a 5′ monophosphate (Figure 1E). Only mutations in the center of the 5′ sensing pocket, R169K and T170V, abolish the ability to distinguish between monophosphate and hydroxyl groups, as shown previously (Callaghan et al., 2005; Jourdan and McDowall, 2008), and they are not activated by the 5′ monophosphate group (Figure 1E); however, they are capable of cleaving 16% and 60%–80% of *ompD*, respectively. The target *ompD* is also cleaved slightly differently by RNase E (1–529) R169K and T170V compared with WT or other RNase E mutants and a product of about 130 nucleotides is formed (Figure 1E). This finding suggests that, when the 5′ binding pocket is not completely functional, the enzyme can still find an alternative way to organize bound substrate at the active site, possibly explaining the viability of cells harboring mutations in the 5′ binding pocket (Garrey et al., 2009).

A Potential Helper Metal Site in the RNase H Domain

Analysis of the crystal structure of *E. coli* RNase E co-crystallized with the sRNA MicC identified a potential metal ion bound in the RNase H domain (Figure 2A) not observed in the previous RNase E structures. The coordinating residues at this site are D26, D28, and from the DNase I domain, D338 and R371. These are expected to make an electrostatically repulsive cluster. The metal is proposed to be magnesium based on the geometry of the interacting carboxylates and backbone carbonyl groups. A structural comparison shows that the active site of *E. coli* RNase H is situated on a distinct site from where the RNase E acidic residues reside (Figure S1), suggesting that this metal does not support cleavage. Notably, the pattern of amino acids at the putative metal binding site, derived as DxDIE (residues 26–30), is highly conserved among RNase E homologs (Figures 2B and S2). The proposed binding of magnesium ion in this pocket may depend on the conformation of RNase E, and is supported by the conformational state associated with MicC binding.

We constructed a triple mutant of RNase E (1–529) D26N, D28N, D338N and tested its activity. These single-atom substitutions are isosteric and retain polar character but result in the loss of electronegativity. Compared to the WT enzyme, the mutant reproduced the same cleavage patterns, but surprisingly showed much greater activity (Figure 2C). The D26, D28, D338 cluster is not close to the hydrolytic center and is unlikely to participate

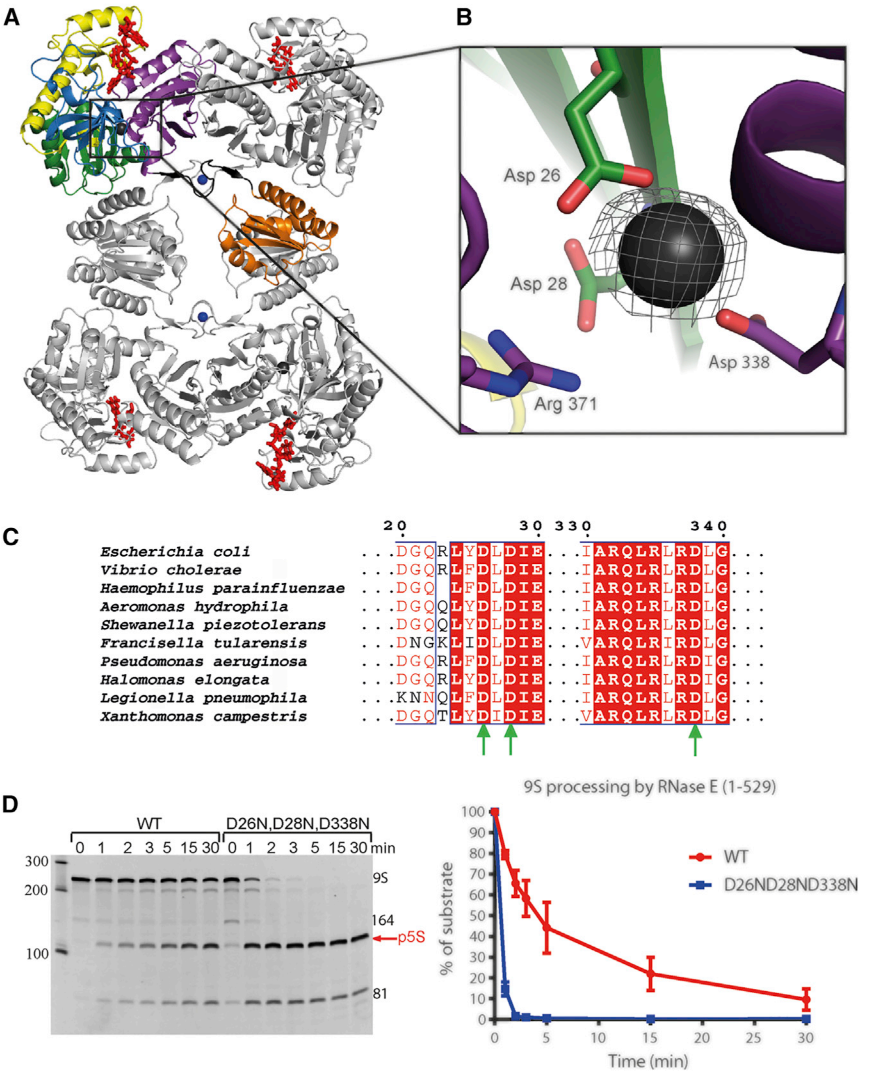

Figure 2. Acidic Pocket in RNase E NTD Coordinates Magnesium Ion and Affects Activity of the Enzyme

(A and B) The conserved acidic region in RNase E (A) and a putative Mg(II) interaction (B). One protomer of RNase E is color coded as in Figure 1. The Fo-Fc anneal-omit map for the magnesium ion was calculated using the phases from the final coordinates and contoured at 2.5 sigma.

(C) Alignment of RNase E catalytic domain from representative species of γ-Proteobacteria. Green arrows mark residues coordinating the metal in the newly identified magnesium binding site.

(D) Substitution of the conserved aspartates with asparagines (RNase E D26N,D28N,D338N) tremendously boosts the activity of the enzyme for processing 9S RNA. In total, 200 nM 9S was incubated with 50 nM RNase E (1–529) wild-type (WT) or the D26N,D28N,D338N mutant. The profiles show averages and SDs from three technical replicates.

directly in the catalytic reaction. Instead, being close to the 5′ binding pocket, it might have an impact on the local electrostatics that could influence the conformational adjustments required for substrate accommodation at the active site or for post-cleavage substrate release.

RNase E Activity Can Be Directed by Structured Elements in the RNA Substrates

To investigate if RNA-specific features, other than 5′ monophosphate, can influence RNase E activity, we devised a simplified *in vitro* assay system comprising of a fragment of the *ompD* mRNA and 12-mer MicC.

The predicted structure of *ompD* contains a stem-loop at the MicC interaction site and an additional stem-loop upstream of the MicC-*ompD* interaction site (Figure 3). An *ompD* fragment that contains both the upstream stem-loop and MicC interaction site is cleaved by RNase E in the absence of MicC 12-mer within the MicC binding region, as was shown before. Addition of MicC guides the enzyme to the cleavage site observed *in vivo* (Bandyra et al., 2012; Pfeiffer et al., 2009). Removal of the upstream stem-loop (50-mer *ompD**) results in almost complete abolishment of the MicC-guided *ompD* cleavage and redirects RNase E to a different site in the newly formed single-stranded region. mRNA with mutations in the stem-loop with increasing base-pairing strength in the stem (50-mer *ompD**2) is still efficiently guided for RNase E cleavage in the presence of 12-mer MicC. Changing the distance between the stem-loop and the MicC binding site did not significantly change RNase E activity (50-mer *ompD**3 and 5); however, when the stem-loops were swapped in position, the sRNA-directed cleavage was lost (50-mer *ompD**4; Figure 3). These results indicate that the activity of RNase E can be directed by *cis*-acting structural elements in the substrate and that these must be accurately positioned for optimal effect, which will be discussed further in the next section.

Crystal Structure of RprA-RNase E (NTD) Complex Explains How Structured Elements in RNA Substrates Are Recognized

To identify additional RNA-enzyme interaction sites within the NTD of RNase E, co-crystallizations were undertaken of the complex of full-length sRNA RprA with the catalytically inactive RNase E (1–510) D303R, D346R. Diffraction data were collected from tens of specimens prepared under different buffer conditions, and the highest resolution obtained was 3.95 Å (Figure 4A, left panel). A molecular replacement solution was found using a model consisting of the core of RNase E (domains RNase H, DNase I, Zn-link and small domain, and 5′ sensors and S1 domains). Despite the low resolution, the electron density

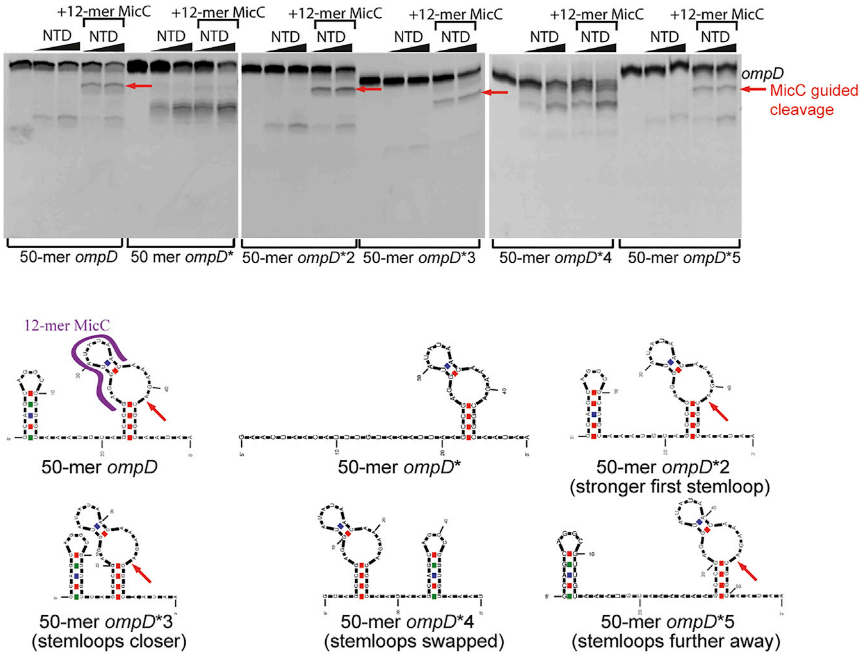

Figure 3. RNase E Cleavage Is Influenced by the Secondary Structures in RNA

50-mer *ompD* RNase E cleavage is guided by 5′P. 12-mer MicC is enhanced by an upstream stem-loop in the mRNA fragment. In the top left schematic of the 50-mer *ompD* substrate, the region of complementarity to the 12-mer MicC seed region is indicated by the purple ribbon. The MicC-induced cleavage position is indicated by a red arrow.

map showed clear features corresponding to a putative double-stranded RNA of about 10 base pairs following building and refinement of the protein component of the complex (Figure 4A, right panel) (Table 1).

Crystals of the complex were dissolved and analyzed on a denaturing RNA gel, revealing the full-length RNA present (data not shown). It is likely that only small portions of the RNA could be modeled into the map due to a combination of low resolution and structural disorder. It was not possible to confidently fit the sequence of the RNA, but based on the size of double-stranded RNA that could be modeled into the density, the duplex is likely to correspond to the 5′ end stem-loop structure, which is the largest of the three predicted stem-loops in RprA (Figure 4A). We obtained lower resolution crystals with another sRNA, SdsR, which also contains structural elements, and one of its stem-loops was found to bind in the same region of RNase E as RprA (data not shown).

The duplex binding region is formed by a surface presented by the RNase H domain of one protomer and the small domain of the partner protomer that forms the principal dimer (i.e., the dimer unit of the dimer of dimers within the tetramer). At the limited resolution of the crystal structure, it is not possible to confidently identify amino acid interactions with the duplex. However, based on the high-resolution structures available for the catalytic domain from our earlier studies, it is possible to formulate a testable hypothesis for which residues are likely to be exposed on the surface of the protein that would be in proximity to the phosphate backbone or bases. Thus, the complex of the RNA duplex with RNase E identified in the crystal structures with RprA and SdsR sRNAs revealed a surface of 8 amino acids of RNase E that could potentially interact with the RNA, namely R3, Q22, H268, Y269, Q270 (from the RNase H domain), K433, R488, and R490 (from the small domain).

Mutations at the Putative Duplex Binding Site Affect Substrate Recognition in the Internal Entry Mode, but Not 5′ Sensing

The amino acids at the proposed duplex recognition surface were replaced in such a way to maximally conserve physicochemical property but weaken or disrupt the proposed interactions with the duplex (R3Q, Q22D, H268S, Y269F, Q270D, K433N, R488Q, and R490Q). Individual mutations at this surface were not found to significantly impact RNA binding or RNase E activity, but the combination of all eight substitutions had the greatest effect and was studied further. This derivative of the duplex recognition surface will hereafter be referred to as the 8x mutant for brevity.

We first tested binding of the sRNA RprA to RNase E (1–529) WT and the 8x mutant. Using bio-layer interferometry, we investigated the binding with mono- and triphosphorylated sRNA in buffer conditions that do not support cleavage (Thompson et al., 2015) (Figure 5A). WT RNase E was capable of binding both 5′ monophosphorylated and 5′ triphosphorylated RprA with similar nanomolar affinity (15.6 ± 4.5 nM and 8.6 ± 7.2 nM, respectively). The 8x mutant, however, was able to detectably bind only to 5′ monophosphorylated sRNA, with lower estimated K_d than WT enzyme (47.0 ± 18.4 nM affinity). When incubated with 5′ triphosphorylated RprA, the recorded response was linear, suggesting very weak binding. Therefore, the 8x mutant has little capacity for interacting with other recognition elements in the absence of a 5′ monophosphate.

We have also tested the activity of the 8x mutant toward 9S rRNA. Compared to WT, the mutant shows limited activity during 30 min incubation at the same concentration when the 9S rRNA had a 5′ triphosphate: the WT RNase E (1–529) processed 98.8% ± 0.3% of the total substrate, whereas the 8x mutant reached 8.9% ± 1.7% at the corresponding time (Figure 5B). 9S is a structured substrate; therefore, the access to the cleavage sites might require recognition of some of the structured elements by the RNase E duplex recognition surface. We tested if providing a 5′ monophosphate would activate the 8x mutant, which is not capable of efficient substrate binding within the identified surface. 5′ monophosphorylated 9S is processed by the 8x mutant, albeit still slower than WT, to form the 5S precursor: the mutant processed 83.1% ± 1.2% of 9S whereas WT

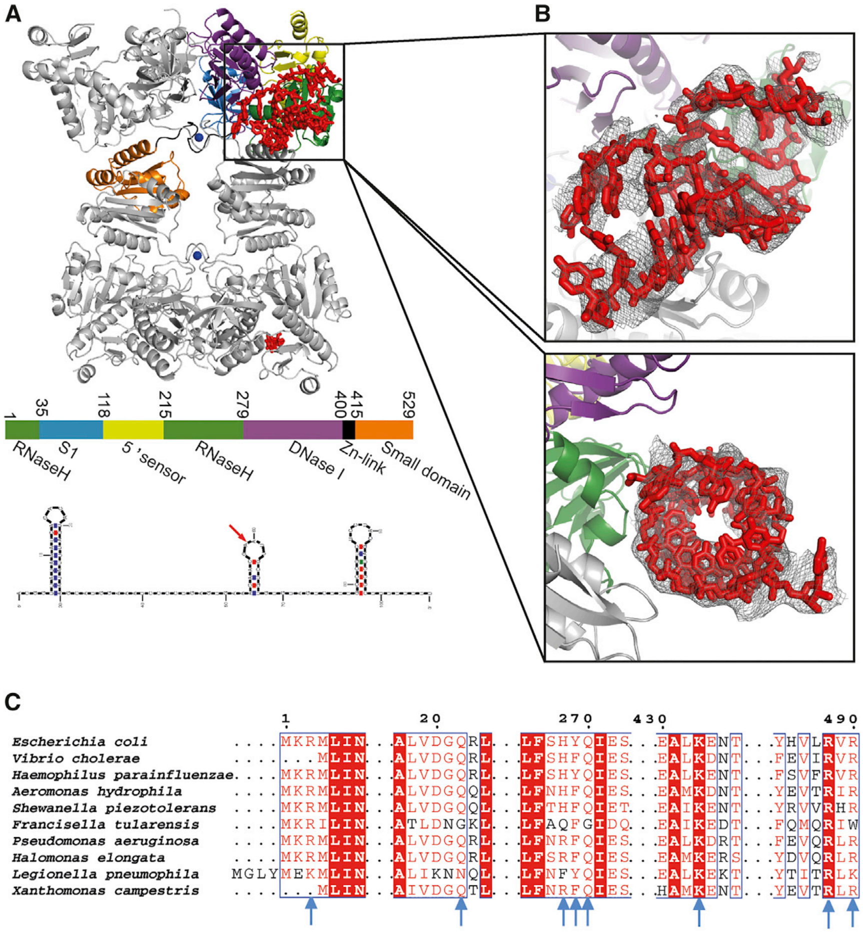

Figure 4. Crystal Structure of the Complex of sRNA RprA with RNase E Catalytic Domain

(A) Refined structure of RNase E (1–511) showing the duplex binding on the surface of the RNase H domain and the small domain of the partner protomer of the principal dimer. The helix is proposed to be from the 5′ stem-loop structure of RprA (bottom panel). The individual subdomains of one protomer are color coded as indicated in the coding bar. RNA is shown in red.

(B) RNA interacting with the duplex binding surface. Fo-Fc anneal-omit electron density map showing putative duplex region and with fitted RNA stem-loop structure was calculated using the final coordinates and contoured at 1.5 sigma.

(C) Alignment of RNase E catalytic domain from representative species of γ-Proteobacteria. Blue arrows mark residues implicated in interaction with RNA structural elements based on the crystal structure of the RNase E NTD/RprA complex.

RNase E (1–529) reached 98.7% ± 0.1%. These results suggest that the RNase E 8x mutant is not capable of efficiently acting in the bypass pathway, which requires recognizing a structural signature in the substrate, but is still active when the substrate has a 5′ monophosphate, which allows access to the cleavage site and substrate binding despite the unavailability of the duplex binding surface. The lower activity of the 8x mutant suggests that the 5′ phosphate recognition pathway and 5′ bypass could cooperate, providing greater activity of the enzyme on a substrate.

Both RNase E WT and 8x mutant were also tested against an artificially constructed substrate, which has two of the RprA non-cleaved stem-loops separated by an A/U-rich segment (Figure 5C, bottom panel; 62-mer). The access to the cleavage site in this short RNA should be possible only upon binding of the structural elements of the triphosphorylated substrate. Similar to 9S rRNA, when the 62-mer has a triphosphate group on the 5′ end, only WT RNase E is capable of cleaving it efficiently, removing 42.4% ± 6.7% after 30 min, whereas the 8x mutant shows a decrease by 2.3% ± 0.8% of the original substrate amount. However, when the 5′ end harbors a monophosphate group, the 8x mutant becomes capable of recognizing the 62-mer and proceeds with the cleavage, processing it similarly to WT enzyme (91.2% ± 3.1% and 97.1% ± 0.3%, respectively).

These results suggest that the limited activity of the 8x mutant is a result of not being able to recognize structural motifs in its RNA substrates. The mutant can still sense the 5′ end of the RNA; however, as the site responsible for 5′ bypass mechanism is not functional, the cleavage that requires recognition of RNA secondary structure does not occur. Similar results were found for the 8x mutant in the context of the RNA degradosome and its subassembly (without PNPase), suggesting that the NTD is sufficient to mediate both bypass and 5′ end recognition (Figure S3).

DISCUSSION

Taking together the data presented here and those previously published, we envisage a general model for different modes by which RNase E can form productive encounter-complexes with RNA substrates. These are summarized schematically in Figure 6. In the 5′ end sensing case (top right panel), a single-stranded accessible 5′ monophosphate and first bases of the 5′ end bind within a sensing pocket, causing appropriate alignment at the active site of the single-stranded region containing the cleavage site. In the internal entry mode (middle right panel), a duplex region of the substrate binds the RNase H/small domain surface. Just as in the case of the 5′ end sensing, the substrate for 5′ bypass must enter the cleavage site in a defined direction with respect to the phosphodiester backbone, because the opposite polarity cannot support the cleavage chemistry. This does not necessarily place a constraint on the location of the duplex either upstream or downstream of the cleavage site because the tetrameric organization of the RNase E allows either location to be compatible with the 5′ bypass mode. For instance,

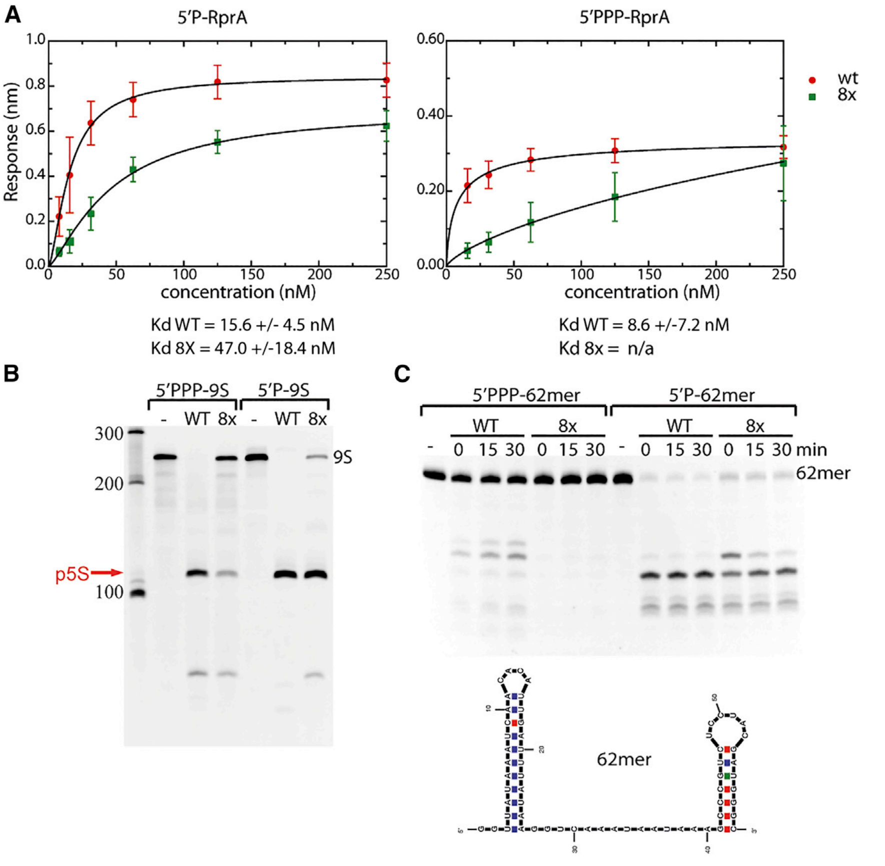

Figure 5. The Duplex Interaction Surface Contributes to RNA Binding and Cleavage

(A) 5′ mono- and triphosphorylated RNA binding by RNaseE (1–529) wild-type (WT) and duplex binding site mutant (8x). The binding experiments were done under conditions in which cleavage is not occurring. The mutant binds 5′ monophosphorylated RprA with about a third the affinity seen for the WT enzyme, but the affinity for triphosphorylated substrate is substantially reduced. The profiles show averages and SDs from three technical replicates.

(B) 9s rRNA (200 nM) with 5′ tri- or monophosphate processing by 1 μM RNaseE (1–529) WT and 8x mutant.

(C) 62-mer RNA (5 μM) with 5′ tri- or monophosphate processing by 1 μM RNaseE (1–529) WT and 8x mutant.

cleavage by the active site proximal to the duplex binding region will be preferred by a downstream duplex, whereas an upstream duplex prefers cleavage by distal active sites. The 5′ bypass and 5′ end sensing modes need not operate strictly independently and in fact could be combined for enhanced activity through recognition of both a stem-loop structure and a 5′ group. The right bottom panels show how 5′ end sensing and internal entry can operate on a single RNA molecule. This mode of interaction has some analogy to the recognition of an internal stem-loop structure and 5′ end cap by the eukaryotic eIF3 (Lee et al., 2015, 2016). For internal entry and 5′ end recognition modes to cooperate, all that is required is an RNA with a stem-loop structure flanked by single-stranded regions that are compatible with the requirements to reach the 5′ sensing pocket at one end and traverse the cleavage site at the other end.

Our data help to explain the finding that the majority of known RNase E cleavage sites are preceded by a stem-loop (Del Campo et al., 2015). For both RNA substrates present in this study, it seems that the stem-loop present upstream of the cleavage site plays an important role in RNase E recognition. The *ompD*-MicC pair appears to be a substrate recognized by both 5′ sensing and 5′ bypass, so requirements for both interaction modes must be met to ensure efficient, guided RNase E cleavage. The *ompD* stem-loop before the MicC binding site is an important signature required together with the 5′ monophosphate delivered on the sRNA to achieve the mRNA cleavage observed *in vivo*. Similarly, from the presented crystal structure of RNase E and RprA it appears that a 5′ stem-loop on sRNA is a key recognition element for the enzyme. The RprA used in the crystallization had a 5′ monophosphate, which is required for its efficient processing by RNase E (data not shown), and is probably engaging in the 5′ binding pocket of the enzyme where it provides allosteric activation.

The identified duplex binding surface comprises a relatively large area, with binding defined between the protein and RNA backbone rather than particular bases. Such a mechanism ensures lack of sequence specificity, but structure specificity: upon stable binding of the stem, if any single-stranded regions are present in the RNA, these can be aligned in the active site. One of the identified amino acids, K433, has been shown before to be engaged in RNA binding by cross-linking studies (Kim et al., 2014). It is possible that the duplex binding site mutations also affects 5′ end recognition pathway; however, the defect observed in the 5′ monophosphorylated substrates degradation by the 8x mutant is modest, therefore this surface is mostly responsible for structured RNA recognition.

It can be envisaged that there are cases in which the 5′ sensing and internal entry modes can be facilitated by guiding regulatory RNAs acting *in trans*. The 5′ activation effect could be achieved by a 5′ monophosphate on an sRNA with a suitably presented 5′ end, as described for MicC (Bandyra et al., 2012). In the internal entry mode, the stem-loop structures could be provided through a guiding sRNA. Both 5′ sensing and duplex recognition could be combined to give a stronger activating effect, but this will depend on the precise location of the free 5′ end, the duplex region, and the single-stranded region of the target. In the case of MicC and *ompD*, both enzyme modes seem to be required for efficient regulation, as the duplex binding surface mutant shows very low activity toward the *ompD* transcript even in the presence of monophosphorylated sRNA, indicating that the stem loop in the mRNA preceding the MicC binding site is an important regulator in this process.

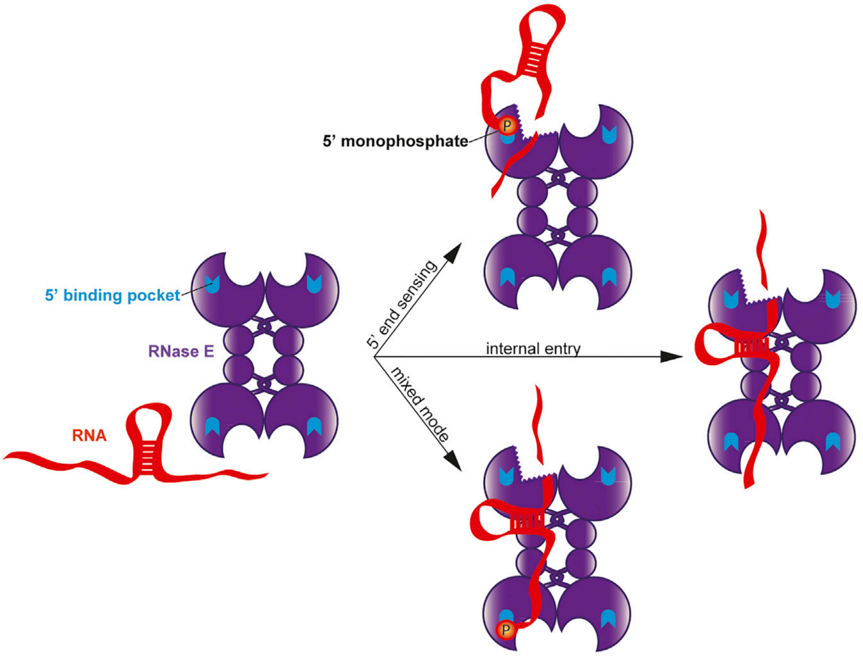

Figure 6. Modes of Interaction of RNase E with RNA

RNase E (purple) can recognize RNA (red) via the 5′ sensing route (top right panel) or direct entry (middle right panel), or can utilize both 5′ end and structural elements (mixed mode, bottom right panel). 5′ end recognition involves 5′ monophosphate binding by the 5′ sensing pocket (blue). The direct entry mode requires RNA fold recognition by the identified RNA duplex binding surface localized between RNase H, DNase I, and small domains. The cleavage of the substrate can occur both upstream and downstream of the structural element, depending on the overall fold and alignment of the RNA on RNase E.

Apart from substrate recognition, some features of the enzyme itself determine its activity. Within the RNase H-like domain of RNase E is a pocket of unknown function that is lined with conserved acidic residues, suggesting that the pocket may be functionally important. The crystal structure of *E. coli* RNase E implies that a magnesium ion could bind here, and while this suggests that the site could support metal-assisted hydrolysis, it seems not to have a catalytic role and even suppresses activity. Replacing the acidic residues with asparagines has no impact on the pattern of sRNA-mediated cleavage of target transcript or the processing of 9S ribosomal RNA precursor. However, the charge-neutralizing changes substantially boost the rate at which substrates are cleaved, indicating that the pocket provides an autoinhibitory function. We note that in the transition from the apo to the activated conformational state of RNase E, this pocket becomes enclosed. Thus, this conserved site might destabilize the active, closed form. While such destabilization might decrease the apparent enzyme activity, it could favor release of cleaved product from the enzyme to suppress potential product inhibition. The conservation of the self-inhibitory motif suggests that RNase E catalytic power has not been optimized in the course of molecular evolution, but has instead been tuned to a lower level that is presumably optimal for fitness within the cellular context.

A picture is emerging of RNase E action in which the enzyme interacts with a substrate through numerous modes, involving binding of duplexes on the RNase H surface, with potential cooperation with 5′ end sensing. Several such duplexes could engage at the four potential binding surfaces of the RNase E tetramer. As duplex binding at one site could influence quaternary structural changes that present the binding surface at a distant site, there is potential for strong cooperativity in RNA interaction. It is also possible that the duplexes can be formed by two RNAs acting *in trans*. These distinct recognition modes could be used when RNase E recruits sRNAs and acts upon sRNA-mRNA pairs.

Numerous studies have revealed that RNase E and the RNA chaperone Hfq work in conjunction in many cases of sRNA-mediated target silencing. Immunoprecipitations have shown that Hfq can physically associate with RNase E *in vivo* (Morita et al., 2004), so one can imagine how this can form an effector complex with sRNAs. RNA sequencing following crosslinking reveals sRNA-mRNA cognate pairs are associated with RNase E (Waters et al., 2017). If the RNase E within the degradosome can capture the Hfq/sRNA complex and present this to interrogate mRNA, then once a partner match is found, the duplex can be displaced from the chaperone for handover to the catalytic domain of RNase E. In principle, double-stranded regions in the sRNA, the target transcript, or the paired sRNA:mRNA could interact with the duplex recognition surface of RNase E to guide the ribonuclease so that it cleaves at defined sites.

STAR★METHODS

Detailed methods are provided in the online version of this paper and include the following:

- KEY RESOURCES TABLE
- CONTACT FOR REAGENT AND RESOURCE SHARING
- METHOD DETAILS
  - RNA preparation
  - RNase E (1-529) and RNase E (1-510) expression and purification
  - RNA degradation assays
  - Crystallization of RNA-protein complex and diffraction data collection
  - Binding rates and affinity measurements
- QUANTIFICATION AND STATISTICAL ANALYSIS
- DATA AND SOFTWARE AVAILABILITY

SUPPLEMENTAL INFORMATION

Supplemental Information includes three figures and can be found with this article online at https://doi.org/10.1016/j.molcel.2018.08.039.

ACKNOWLEDGMENTS

This work is supported by the Wellcome Trust (200873/Z/16/Z). J.M.W. was supported by an Erasmus scholarship. We thank the staff of Diamond Light Source (stations IO4 and I24) and Soleil (Proxima 2A) for access to facilities. We thank Tom Dendooven and Steven Hardwick for helpful suggestions and advice. We thank Gigi Storz, Kenny McDowall, Justin Clarke, and Kirans Sabharwal for helpful comments. We thank A.J. Carpousis and Kai Papenfort for kindly providing reagents. We are grateful to Tristan Croll for improving the refinement of the crystal structures and for helpful comments.

AUTHOR CONTRIBUTIONS

K.J.B. and B.F.L. designed the experiments and analyzed the results. K.J.B., J.M.W., and B.F.L. conducted the experiments and analyzed data. B.F.L. and K.J.B. wrote the manuscript.

DECLARATION OF INTERESTS

The authors have no interests to declare.

Received: March 26, 2018
Revised: July 21, 2018
Accepted: August 24, 2018
Published: September 27, 2018

REFERENCES

Adams, P.D., Afonine, P.V., Bunkóczi, G., Chen, V.B., Davis, I.W., Echols, N., Headd, J.J., Hung, L.-W., Kapral, G.J., Grosse-Kunstleve, R.W., et al. (2010). PHENIX: a comprehensive Python-based system for macromolecular structure solution. Acta Crystallogr. D Biol. Crystallogr. *66*, 213–221.

Aït-Bara, S., Carpousis, A.J., and Quentin, Y. (2015). RNase E in the γ-Proteobacteria: conservation of intrinsically disordered noncatalytic region and molecular evolution of microdomains. Mol. Genet. Genomics *290*, 847–862.

Bandyra, K.J., Said, N., Pfeiffer, V., Górna, M.W., Vogel, J., and Luisi, B.F. (2012). The seed region of a small RNA drives the controlled destruction of the target mRNA by the endoribonuclease RNase E. Mol. Cell *47*, 943–953.

Bandyra, K.J., Bouvier, M., Carpousis, A.J., and Luisi, B.F. (2013). The social fabric of the RNA degradosome. Biochim. Biophys. Acta *1829*, 514–522.

Bouvier, M., and Carpousis, A.J. (2011). A tale of two mRNA degradation pathways mediated by RNase E. Mol. Microbiol. *82*, 1305–1310.

Callaghan, A.J., Aurikko, J.P., Ilag, L.L., Günter Grossmann, J., Chandran, V., Kühnel, K., Poljak, L., Carpousis, A.J., Robinson, C.V., Symmons, M.F., and Luisi, B.F. (2004). Studies of the RNA degradosome-organizing domain of the Escherichia coli ribonuclease RNase E. J. Mol. Biol. *340*, 965–979.

Callaghan, A.J., Marcaida, M.J., Stead, J.A., McDowall, K.J., Scott, W.G., and Luisi, B.F. (2005). Structure of Escherichia coli RNase E catalytic domain and implications for RNA turnover. Nature *437*, 1187–1191.

Carpousis, A.J. (2007). The RNA degradosome of *Escherichia coli*: an mRNA-degrading machine assembled on RNase E. Annu. Rev. Microbiol. *61*, 71–87.

Chao, Y., Li, L., Girodat, D., Förstner, K.U., Said, N., Corcoran, C., Śmiga, M., Papenfort, K., Reinhardt, R., Wieden, H.J., et al. (2017). In vivo cleavage map illuminates the central role of RNase E in coding and non-coding RNA pathways. Mol. Cell *65*, 39–51.

Christiansen, J. (1988). The 9S RNA precursor of Escherichia coli 5S RNA has three structural domains: implications for processing. Nucleic Acids Res. *16*, 7457–7476.

Clarke, J.E., Kime, L., Romero A, D., and McDowall, K.J. (2014). Direct entry by RNase E is a major pathway for the degradation and processing of RNA in Escherichia coli. Nucleic Acids Res. *42*, 11733–11751.

Cormack, R.S., and Mackie, G.A. (1992). Structural requirements for the processing of Escherichia coli 5 S ribosomal RNA by RNase E in vitro. J. Mol. Biol. *228*, 1078–1090.

Croll, T.I. (2018). ISOLDE: a physically realistic environment for model building into low-resolution electron-density maps. Acta Crystallogr. D Struct. Biol. *74*, 519–530.

Dar, D., and Sorek, R. (2018). Extensive reshaping of bacterial operons by programmed mRNA decay. PLoS Genet. *14*, e1007354.

Del Campo, C., Bartholomäus, A., Fedyunin, I., and Ignatova, Z. (2015). Secondary structure across the bacterial transcriptome reveals versatile roles in mRNA regulation and function. PLoS Genet. *11*, e1005613.

Dimastrogiovanni, D., Fröhlich, K.S., Bandyra, K.J., Bruce, H.A., Hohensee, S., Vogel, J., and Luisi, B.F. (2014). Recognition of the small regulatory RNA RydC by the bacterial Hfq protein. eLife *3*, https://doi.org/10.7554/eLife.05375.

Emsley, P., Lohkamp, B., Scott, W.G., and Cowtan, K. (2010). Features and development of Coot. Acta Crystallogr. D Biol. Crystallogr. *66*, 486–501.

Garrey, S.M., and Mackie, G.A. (2011). Roles of the 5′-phosphate sensor domain in RNase E. Mol. Microbiol. *80*, 1613–1624.

Garrey, S.M., Blech, M., Riffell, J.L., Hankins, J.S., Stickney, L.M., Diver, M., Hsu, Y.H.R., Kunanithy, V., and Mackie, G.A. (2009). Substrate binding and active site residues in RNases E and G: role of the 5′-sensor. J. Biol. Chem. *284*, 31843–31850.

Ghora, B.K., and Apirion, D. (1978). Structural analysis and in vitro processing to p5 rRNA of a 9S RNA molecule isolated from an rne mutant of E. coli. Cell *15*, 1055–1066.

Huang, H., Liao, J., and Cohen, S.N. (1998). Poly(A)- and poly(U)-specific RNA 3′ tail shortening by E. coli ribonuclease E. Nature *391*, 99–102.

Jourdan, S.S., and McDowall, K.J. (2008). Sensing of 5′ monophosphate by Escherichia coli RNase G can significantly enhance association with RNA and stimulate the decay of functional mRNA transcripts in vivo. Mol. Microbiol. *67*, 102–115.

Kaberdin, V.R., Walsh, A.P., Jakobsen, T., McDowall, K.J., and von Gabain, A. (2000). Enhanced cleavage of RNA mediated by an interaction between substrates and the arginine-rich domain of E. coli ribonuclease E. J. Mol. Biol. *301*, 257–264.

Khemici, V., Poljak, L., Luisi, B.F., and Carpousis, A.J. (2008). The RNase E of Escherichia coli is a membrane-binding protein. Mol. Microbiol. *70*, 799–813.

Kim, D., Song, S., Lee, M., Go, H., Shin, E., Yeom, J.H., Ha, N.C., Lee, K., and Kim, Y.H. (2014). Modulation of RNase E activity by alternative RNA binding sites. PLoS ONE *9*, e90610.

Kime, L., Jourdan, S.S., Stead, J.A., Hidalgo-Sastre, A., and McDowall, K.J. (2010). Rapid cleavage of RNA by RNase E in the absence of 5′ monophosphate stimulation. Mol. Microbiol. *76*, 590–604.

Kime, L., Clarke, J.E., Romero A, D., Grasby, J.A., and McDowall, K.J. (2014). Adjacent single-stranded regions mediate processing of tRNA precursors by RNase E direct entry. Nucleic Acids Res. *42*, 4577–4589.

Koslover, D.J., Callaghan, A.J., Marcaida, M.J., Garman, E.F., Martick, M., Scott, W.G., and Luisi, B.F. (2008). The crystal structure of the Escherichia coli RNase E apoprotein and a mechanism for RNA degradation. Structure *16*, 1238–1244.

Lee, A.S.Y., Kranzusch, P.J., and Cate, J.H.D. (2015). eIF3 targets cell-proliferation messenger RNAs for translational activation or repression. Nature *522*, 111–114.

Lee, A.S.Y., Kranzusch, P.J., Doudna, J.A., and Cate, J.H.D. (2016). eIF3d is an mRNA cap-binding protein that is required for specialized translation initiation. Nature *536*, 96–99.

Mackie, G.A. (1998). Ribonuclease E is a 5′-end-dependent endonuclease. Nature *395*, 720–723.

Mackie, G.A. (2013). RNase E: at the interface of bacterial RNA processing and decay. Nat. Rev. Microbiol. *11*, 45–57.

Majdalani, N., Hernandez, D., and Gottesman, S. (2002). Regulation and mode of action of the second small RNA activator of RpoS translation, RprA. Mol. Microbiol. *46*, 813–826.

Marcaida, M.J., DePristo, M.A., Chandran, V., Carpousis, A.J., and Luisi, B.F. (2006). The RNA degradosome: life in the fast lane of adaptive molecular evolution. Trends Biochem. Sci. *31*, 359–365.

McDowall, K.J., Lin-Chao, S., and Cohen, S.N. (1994). A+U content rather than a particular nucleotide order determines the specificity of RNase E cleavage. J. Biol. Chem. *269*, 10790–10796.

McDowall, K.J., Kaberdin, V.R., Wu, S.W., Cohen, S.N., and Lin-Chao, S. (1995). Site-specific RNase E cleavage of oligonucleotides and inhibition by stem-loops. Nature *374*, 287–290.

Morita, T., Kawamoto, H., Mizota, T., Inada, T., and Aiba, H. (2004). Enolase in the RNA degradosome plays a crucial role in the rapid decay of glucose transporter mRNA in the response to phosphosugar stress in Escherichia coli. Mol. Microbiol. *54*, 1063–1075.

Papenfort, K., Espinosa, E., Casadesús, J., and Vogel, J. (2015). Small RNA-based feedforward loop with AND-gate logic regulates extrachromosomal DNA transfer in *Salmonella*. Proc. Natl. Acad. Sci. USA *112*, E4772–E4781.

Pfeiffer, V., Papenfort, K., Lucchini, S., Hinton, J.C.D., and Vogel, J. (2009). Coding sequence targeting by MicC RNA reveals bacterial mRNA silencing downstream of translational initiation. Nat. Struct. Mol. Biol. *16*, 840–846.

Soper, T., Mandin, P., Majdalani, N., Gottesman, S., and Woodson, S.A. (2010). Positive regulation by small RNAs and the role of Hfq. Proc. Natl. Acad. Sci. USA *107*, 9602–9607.

Thompson, K.J., Zong, J., and Mackie, G.A. (2015). Altering the divalent metal ion preference of RNase E. J. Bacteriol. *197*, 477–482.

Waters, S.A., McAteer, S.P., Kudla, G., Pang, I., Deshpande, N.P., Amos, T.G., Leong, K.W., Wilkins, M.R., Strugnell, R., Gally, D.L., et al. (2017). Small RNA interactome of pathogenic *E. coli* revealed through crosslinking of RNase E. EMBO J. *36*, 374–387.

Winn, M.D., Ballard, C.C., Cowtan, K.D., Dodson, E.J., Emsley, P., Evans, P.R., Keegan, R.M., Krissinel, E.B., Leslie, A.G., McCoy, A., et al. (2011). Overview of the CCP4 suite and current developments. Acta Crystallogr. D Biol. Crystallogr. *67*, 235–242.

STAR★METHODS

KEY RESOURCES TABLE

REAGENT or RESOURCE	SOURCE	IDENTIFIER
**Bacterial and Virus Strains**		
BL21 (DE3) for protein overexpression	ATCC	BL21 (DE3)
**Chemicals, Peptides, and Recombinant Proteins**		
RNase E (1-529)	This study	RNase E (1-529)
RNase E (1-511)	This study	RNase E (1-511)
RNase E (1-529) R3Q, Q22D, H268S, Y269F, Q270D, K433N, R488Q, R490Q	This study	RNase E (1-529) 8x mutant
RNase E (1-529) D26N, D28N, D338N	This study	RNase E (1-529) D26N, D28N, D338N
RNase E R141Q	This study	RNase E (1-529) R141Q
RNase E R142Q	This study	RNase E (1-529) R142Q
RNase E R373K	This study	RNase E (1-529) R373K
RNase E R373Q	This study	RNase E (1-529) R373Q
RNase E R169K	This study	RNase E (1-529) R169K
RNase E T170V	This study	RNase E (1-529) T170V
RNase E (1-850) R3H, Q22D, H268S, Y269F, Q270D, K433N, R488Q, R490Q	This study	RNase E (1-850)
RNase E R3H, Q22D, H268S, Y269F, Q270D, K433N, R488Q, R490Q	This study	RNase E
**Deposited Data**		
Crystallographic data and model, RNase E/MicC	This study	PDB: 5F6C
Crystallographic data and model, RNase E/RprA	This study	PDB: 6G63
Images of gels, Mendeley Data	This study	https://doi.org/10.17632/crmm6ccgy4.1
**Oligonucleotides**		
GTTTTTTTTTTAATACGACTCACTATTACGGTTATAA ATCAACACATTG	This study	RprA forward
AAAAAAAAGCCCATCGTAGGAG	This study	RprA reverse
GTTTTTAATACGACTCACTATAGAAGCTGTTTTGGC GGATGAGAG	This study	9S forward
CGAAAGGCCCAGTCTTTCGACTGAGC	This study	9S reverse
MicC seed region connected through a tetranucleotide loop with a fragment of the *ompD* target encompassing the seed complementary sequence and the RNase E cleavage site GUUAUAUGCCUUCUUGGAGGUAUAUA ACAAAGmAmC	This study	MicC-ompD fusion
GUUAUAUGCCUU	Bandyra et al., 2012	12-mer MicC seed
ttttctcgagttaatacgactcactatagGCCATTGACAAACGCC TCGTTTAACAATGG	Bandyra et al., 2012	ompD forward
CGTGAACTTTACCGTACAGATCCAGTTTATTGCCG	Bandyra et al., 2012	ompD reverse
GCAGCAGGCGUUGUAAAUGCAGCCGAGGUAUAUA ACAAAGACGGCAAUAA	This study	50-mer *ompD*
GGUUAUAAAUCAACACAUUGAUUUAUAAGGUCAAA UAAUAAAGCCCGUCUCCUACGAUGGGC	This study	62-mer
**Recombinant DNA**		
Expression plasmid for RNase E 1-529 wild type	Callaghan et al., 2005	pRNE529-N
Expression plasmid for RNase E 1-510 wild type	This study	pRNE510-N
Expression plasmid for RNase E 1-529 R3Q, Q22D, H268S, Y269F, Q270D, K433N, R488Q, R490Q	This study	pRNE529 8x mutant

(*Continued on next page*)

Continued		
REAGENT or RESOURCE	SOURCE	IDENTIFIER
Expression plasmid for RNase E (1-529) D26N, D28N, D338N	This study	pRNE529 D26N, D28N, D338N
Expression plasmid for RNase E R141Q	This study	pRNE529 R141Q
Expression plasmid for RNase E R142Q	This study	pRNE529 R142Q
Expression plasmid for RNase E R373Q	This study	pRNE529 R373Q
Expression plasmid for RNase E R169K	This study	pRNE529 R169K
Expression plasmid for RNase E T170V	This study	pRNE529 T170V
Expression plasmid for RNase E 1-850 R3H, Q22D, H268S, Y269F, Q270D, K433N, R488Q, R490Q	This study	pRNE850 8x mutant
Expression plasmid for RNase E R3H, Q22D, H268S, Y269F, Q270D, K433N, R488Q, R490Q	This study	pRNE 8x mutant
Software and Algorithms		
CCP4 crystallographic suite	Winn et al. (2011)	CCP4
PHENIX	Adams et al. (2010)	PHENIX
Profit	Quantum Soft, Switzerland	Profit
Octet Data Analysis software	ForteBio	Octet Data Analysis
GeneSnap and GeneTools	Syngene	GeneSnap, GeneTools
PyMOL	DeLano Scientific	PyMOL
WinCoot	Emsley et al. (2010)	WinCoot
ISOLDE	Croll (2018)	ISOLDE

CONTACT FOR REAGENT AND RESOURCE SHARING

Further information and requests for resources and reagents should be directed to and will be fulfilled by the Lead Contact, Ben Luisi (bfl20@cam.ac.uk).

METHOD DETAILS

RNA preparation

Plasmids carrying 9S and RprA genes were provided by A.J. Carpousis and K. Papenfort, respectively. Forward PCR primers were designed in a way to add promoter sequence recognized by T7 RNA polymerase. PCR products were used as *In Vitro* Transcription (IVT) templates. IVT was carried out according to standard protocol with addition of 3% DMSO (v/v), followed by DNA template digestion with TURBO DNase (Thermo Fisher). For synthesis of 5′ monophosphorylated RNA, five-fold excess of rAMP or rGMP over rATP or rGTP was used for RprA and 9S respectively to cap the product (Bandyra et al., 2012). Synthesized RNAs were purified on 4% (9S) or 6% (RprA) polyacrylamide gel containing 7.5 M urea (National Diagnostics). The bands were visualized with UV shadowing and excised, and RNAs were eluted from gel slices by overnight electroelution (100V, EluTrap, Whatman). The *ompD* RNA was prepared as previously described (Bandyra et al., 2012).

**Primers used for IVT template preparation**		
Primer name		Sequence 5′ → 3′
RprA	For	GTTTTTTTTTTAATACGACTCACTATTACGGTTATAAATCAACACATTG
	Rev	AAAAAAAAGCCCATCGTAGGAG
9S	For	GTTTTTAATACGACTCACTATAGAAGCTGTTTTGGCGGATGAGAG
	Rev	CGAAAGGCCCAGTCTTTCGACTGAGC

RNase E (1-529) and RNase E (1-510) expression and purification

Mutants of RNase E (1-529) were prepared by two, successive PCR reactions. In the first step, two fragments were PCR-amplified using the wild-type RNase E gene as a template: one using forward primer complementary to the 5′ end of the gene and reverse primer introducing the mutation; the second reaction amplified a DNA fragment using reverse primer complementary to the 3′ end

of the gene and forward primer introducing the mutation (forward and reverse primers introducing the mutation were complementary). PCR products were resolved on 1% low melting point agarose gels, the bands of interest were excised and after melting the matrix by incubation at 70°C, mixed in one PCR reaction which amplified the whole gene with mutation using forward and reverse primers complementary to the 5′ and 3′ end of the gene, respectively. The product of the last PCR was digested with NdeI and BamHI (NEB), resolved on a low melting point agarose gel, and the gel band was directly ligated with T4 ligase (NEB) into a pET16 plasmid, which had been digested with the same restriction enzymes and dephosphorylated with CIP (NEB) according to the manufacturer's instructions.

RNase E (1-529) and (1-510) wild-type and all the mutants were prepared as previously described (Callaghan et al., 2005). In brief, BL21(DE3) cells harboring the plasmid pRne529-N or pRne510-N, which encodes RNase E catalytic domain with N-terminal His-tag, were grown in 2 × YT media (Formedium) supplemented with 100 μg/mL carbenicillin in dimpled flasks to facilitate aeration at 37°C. At OD_{600} = 0.6, the cultures were induced with 1 mM IPTG, and three hours later, cells were harvested by centrifugation, resuspended in buffer A (20 mM Tris-HCl pH 7.9, 500 mM NaCl, 5 mM imidazole, protease inhibitor cocktail tablet (Roche)), and passed three times through an EmulsiFlex-05 cell disruptor (Avestin) at 10-15 kbar. The lysate was supplemented with DNase I (1 μg/mL), clarified by centrifugation (4°C, 30 min, 37500 *g*) and loaded on a 5ml HiTrap Chelating HP column (GE Healthcare) charged with nickel ions. Proteins were eluted by imidazole gradient (buffer A supplemented with 0.5 M imidazole).

When the protein was contaminated with RNA after the nickel step, an additional purification step was added using a HiTrap Heparin column (GE Healthcare) with buffer A (20 mM Tris-HCl pH 7.9, 30 mM NaCl, 1 mM $MgCl_2$) for loading and a gradient of buffer B (buffer A supplemented with 2 M NaCl) for elution; or HP Butyl Sepharose column (GE Healthcare) using buffer C (25 mM Tris pH 7.5, 50 mM NaCl, 25 mM KCl, 1 M $(NH_4)_2SO_4$) for loading and a gradient of buffer D (50 mM Tris pH 7.5, 50 mM NaCl, 10 mM KCl, 0.02% B-dodecylmaltoside, 5% glycerol) for elution.

Fractions enriched in RNaseE catalytic domain were pooled, concentrated to 1 mL with 15 mL Amicon Ultra 30,000 MWCO concentrator (Millipore) and loaded on to a Sephadex 200 gel filtration column (GE Healthcare) equilibrated with buffer containing 20 mM HEPES pH 7.0, 500 mM NaCl, 10 mM $MgCl_2$, 0.5 mM EDTA, 0.5 mM TCEP and 5% vol/vol glycerol. Eluted fractions were analyzed by SDS–PAGE and those containing purified RNase E catalytic domain were flash frozen with liquid nitrogen and stored at –80°C. The protein concentration was determined spectroscopically using a NanoDrop ND-1000 spectrophotometer (Thermo Scientific) and a γ_{280nm} extinction coefficient of 28880 $M^{-1}cm^{-1}$.

RNA degradation assays

Assays were carried out in RB buffer (25 mM Tris-HCl pH 7.5, 50 mM NaCl, 50 mM KCl, 10 mM $MgCl_2$, 1 mM DTT, 0.5 U/μL RNase OUT) at 37°C. Time course reactions were stopped at indicated time points by addition of proteinase K in PK buffer (100 mM, 12.5 mM EDTA, 150 mM NaCl, 1% wt/v SDS). Samples were incubated at 50°C for 20 min to digest proteins. RNA loading dye (Thermo Fisher) was added to samples which were denatured (98°C, 2 min) and loaded onto polyacrylamide gels containing 7.5 M urea. Gels were stained with SybrGOLD (Thermo Fisher) and visualized under UV light using GeneSnap software and quantified using GeneTools (Syngene). The assays were carried out in triplicate.

Crystallization of RNA-protein complex and diffraction data collection

RNA samples were annealed (50°C, 2 min) and cooled slowly to room temperature. Inactive RNase E (1-529) D303R, D346R was incubated with RNA (1:1 molar ratio) in crystallization buffer CB (20 mM Tris-HCl pH 7.9, 500 mM NaCl, 10 mM $MgCl_2$, 0.5 mM EDTA with freshly added 10 mM dithiothreitol) for 10 min at room temperature. RNA-protein complex was applied to PD10 buffer exchange column (GE Healthcare) equilibrated with crystallization buffer CB and eluted with crystallization buffer CB. Sample was concentrated using 10kDa Amicon filters (Millipore) to the final complex concentration of 254 μM. Crystallization trials were carried out by sitting drop vapor diffusion method using 96-well plates with commercially available screening condition sets. NTD-RprA complex crystallized after 5-8 days and continued to grow until day 13 in following conditions: 0.1 M KCl, 0.01 M $MgCl_2$, 0.05 M Tris-HCl pH 8.5 and 30% (v/v) PEG 400 at 20°C. Crystals were plunge frozen in liquid nitrogen. Diffraction data were collected at Diamond Light Source (Oxfordshire, UK) and at Soleil Synchrotron (France). For diffraction data processing, molecular replacement and initial refinement following software was used: iMosflm, PhaserMR, Molrep, Refmac5 (CCP4 suite). In the first step of molecular replacement, the search object was a dimer of the RNase E core without the S1/5′ sensor domains, and the molecular replacement solution correctly generate the tetramer through self-complementary interactions of the small domains. The next step used the S1'/5 sensor domain as a search object, and these were positioned consistent with the location of the termini. The maps indicated high degree of disorder of these domains, and they were refined using TLS fitting. The early refined map from this model identified features for a RNA duplex that was included in the model, as well as short fragments of single stranded 5′ monophosphate RNA at two of the 5′ sensing sites that coincided with the position of the RNA seen in higher resolution models. The model was then remodelled and refined using PHENIX (Adams et al., 2010) and ISOLDE (Croll, 2018). Structural figures were made using PyMOL (DeLano Scientific) and WinCoot (Emsley et al., 2010).

Binding rates and affinity measurements

Kinetic measurements with Bio-Layer Interferometry were performed using an Octet RED96 equipped with Streptavidin sensors (ForteBio, UK) on 96-well plates. The experiment was performed in the binding buffer (25 mM HEPES pH 7.0, 150 mM NaCl,

100 mM KCl, 10 mM $CaCl_2$, 0.5 mM TCEP), which was also used to prepare all dilutions, for dissociation and neutralisation. An RprA sRNA was labeled with biotin on the 3′ end by ligation with U-biotin (Dharmacon). RNA was immobilised on the biosensors that were subsequently submerged into 0.8 μM solution of maltose binding protein (MBP) labeled with biotin. The binding of wild-type and mutant RNase E (1-529) were assayed at 0, 15.62, 31.25, 62.5, 125, 250 and 500 nM protein over 300 s. The dissociation was monitored over 300 s and was followed by regeneration of the sensors using 1 M $MgCl_2$. Another set of tips was saturated with MBP-biotin and the measurements were then repeated for all RNase E concentration series. The data were analyzed using the Octet Data Analysis software and plotted with ProFit (Quantum Soft, Switzerland) as described by Dimastrogiovanni et al. (2014).

QUANTIFICATION AND STATISTICAL ANALYSIS

Statistical details of experiments can be found in the results section for the RNA degradation assays, and in Table 1 for the crystallographic data.

DATA AND SOFTWARE AVAILABILITY

The crystallographic structure factors and refined coordinates have been deposited with the PDB (PDB: 5F6C and 6G63). The image files for the RNA degradation assays are deposited with Mendeley Data at https://doi.org/10.17632/crmm6ccgy4.1.

Molecular Cell, Volume *72*

Supplemental Information

Substrate Recognition and Autoinhibition in the Central Ribonuclease RNase E

Katarzyna J. Bandyra, Joanna M. Wandzik, and Ben F. Luisi

SUPPLEMENTARY INFORMATION

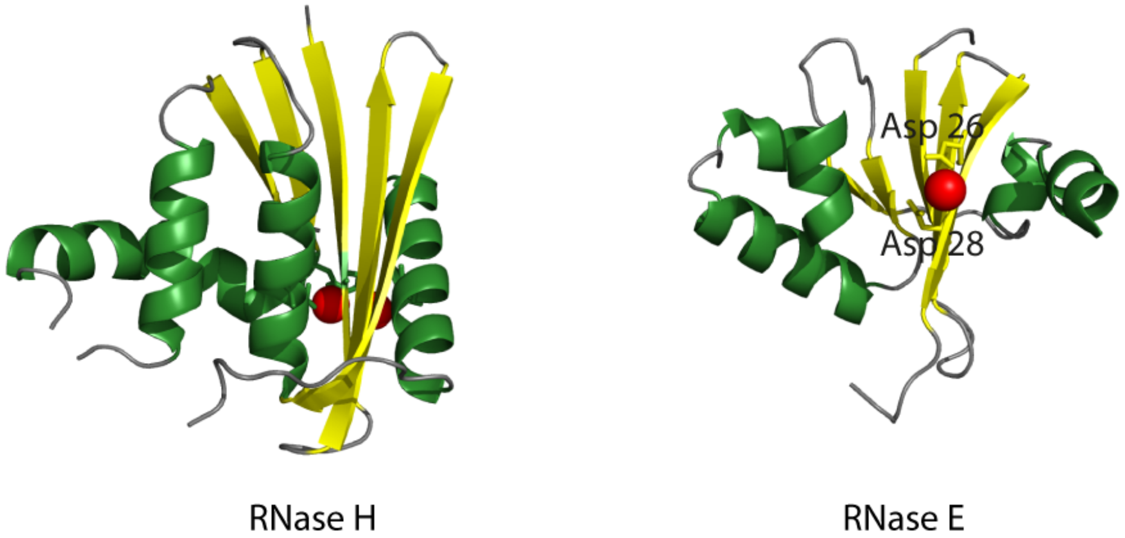

Figure S1. Related to Figure 2. Comparison of E. coli RNase H (1G15) and RNase H domain from RNase E-MicC structure. Bound metal ions (two manganese ions for RNase H and one magnesium ion for RNase E) are shown as red spheres. The acidic residues coordinating metal ions are shown as sticks.

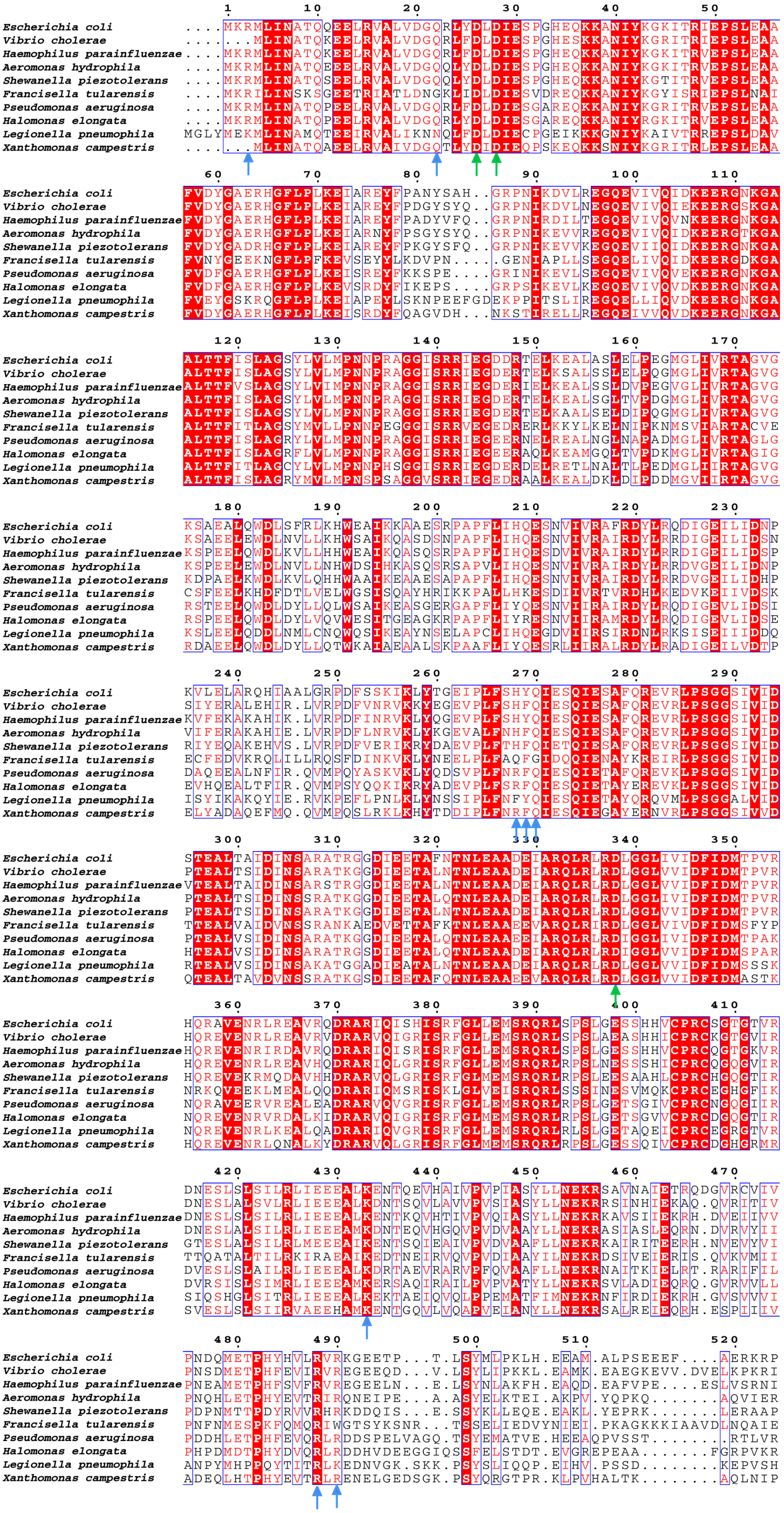
Escherichia coli
Vibrio cholerae
Haemophilus parainfluenzae
Aeromonas hydrophila
Shewanella piezotolerans
Francisella tularensis
Pseudomonas aeruginosa
Halomonas elongata
Legionella pneumophila
Xanthomonas campestris

Figure S2. Related to Figure 2. Alignment of RNase E catalytic domain from representative species of γ-Proteobacteria. Green arrows mark residues coordinating magnesium ion in the newly identified magnesium binding site; blue arrows mark residues implicated in interaction with RNA structural elements based on the crystal structure of the RNase E NTD/RprA complex.

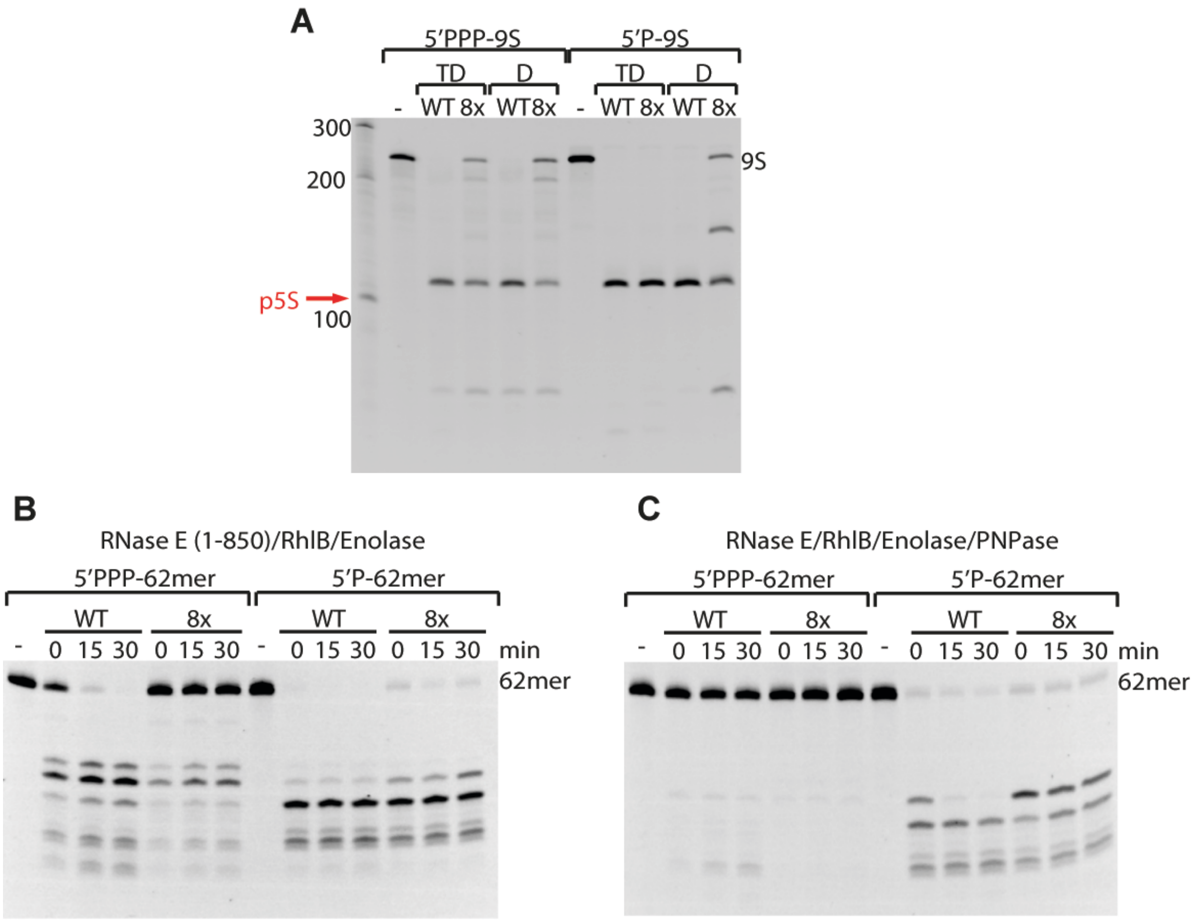

Figure S3. Related to Figure 5. The duplex interaction surface contributes to RNA binding and cleavage. A) 9S rRNA (200 nM) with 5' tri- or monophosphate processing by 100 nM RNase E (1-850)/RhlB/Enolase (TD, truncated degradosome) and 15 nM full length RNase E/RhlB/Enolase/PNPase (D, degradosome), wild type and 8x mutants (R3H, Q22D, H268S, Y269F, Q270D, K433N, R488Q, R490Q). C) 62-mer RNA (5μM) with 5' tri- or monophosphate processing by 200 nM RNase E (1-850)/RhlB/Enolase and 32 nM full length RNase E/RhlB/Enolase/PNPase, wild type and 8x mutants.
